# Paving the way toward better peer review

**DOI:** 10.1038/ncomms13625

**Published:** 2016-11-10

## Abstract

We are committed to support our reviewers with all relevant information needed to draft their reports

Despite the growth of post-publication forums such as a Faculty of 1000, scientific blogs and online commenting, editor-mediated peer review remains the primary means by which science is evaluated ahead of publication. The peer review process critically relies on the report that is provided by the reviewers and that the editors consider in detail and discuss prior to making a decision on the paper. In order to improve the quality of the reports in the evaluation of the paper, *Nature Communications* has made a number of changes to the instructions for reviewers that we hope will assist in the evaluation of the paper and eventually improve the quality of the science that is published.

The main goal of the reviewer report is to provide an objective and thorough evaluation of the study, considering both the technical aspects and the potential impact in the context of the published literature. In an attempt to help reviewers in composing their report, we have replaced the previous list of questions with broader questions about the claims of the manuscript, data supporting those claims, the interest of colleagues in the field and whether the paper will influence thinking in the specific field.

There are a number of principles that in our view are important to the drafting of a reviewer report, and to the subsequent discussion of a paper in post-discussion forums and conferences. First and foremost, reviewers need to have all the information that they need in order to properly evaluate a paper. In particular, all relevant aspects of a study, including the methods, need to be clearly described. Data should be deposited in repositories where possible and made available to reviewers. To help with this and to ensure the transparency and reproducibility of this published output, Nature journals, including *Nature Communications*, have introduced mandatory reporting checklists in a number of research areas that detail statistical information, experimental design and reagents, reproducibility of experiments, and compliance with editorial policies. These checklists are passed on to the reviewers to assist them in the evaluation of the methodology, and ensure the robustness of the work. Importantly, these checklists also help editors ensure that all the relevant methodological information appears in the final published paper.

***Nature Communications* has made a number of changes to the instructions for reviewers that we hope will assist in the evaluation of the paper and eventually improve the quality of the science that is published.**

Secondly, we are dependent on the advice of our reviewers when making decision on a paper after peer review. Our aim at *Nature Communications* is to select the papers based on the significance of the science presented, even if they may sometimes appeal only to a specialized community or to a small section of our wide readership. With this in mind we ask that reviewers not only evaluate the technical aspects of the study but also give their opinion on the potential impact of the manuscript in the context of the published literature. While ultimately an editorial decision, our criteria for publication are shaped by reviewer advice and feedback from the scientific community.

When composing a report we ask that the reviewer carefully assess if the data presented in the manuscript support the main conclusions of the work and suggest how the authors could improve the manuscript and fill eventual gaps. The reviewer report should aim to assess the science presented within the manuscript and not merely list a series of experiments aimed at expanding the scope of the manuscript.

We aim for a quick turnaround for our authors, so we encourage the report to be submitted in a timely fashion to avoid unnecessary delays. Reviewers should be aware that by accepting to review on a colleagues' article, they imply availability to review following versions of the manuscript. Without this policy it would be difficult to maintain consistency in subsequent review cycles. It is important to raise concerns regarding the novelty of the findings presented in a manuscript at the first round of review; successive rounds should mostly focus on evaluating the extent to which the concerns previously raised have been addressed, unless additional very serious concerns that undermine the central conclusions of the paper arise during revision. Reviewers can decide to add a comment that is not disclosed to the authors but made visible to the editors. To make the process as transparent as possible we ask that any recommendations given to the editor are consistent with the advice given to the author and that all the technical concerns are included in the comments made visible to the authors.

The decision on whether to proceed with publication is taken by the editorial team after discussing the reviewers' reports. Although we appreciate that the reviewer's time is precious, we would invite reviewers to write succinct but comprehensive reports; in fact, given the key role of the reviewers' input, longer rather than short and incomplete reports are a tremendous help in reaching an editorial decision. Even if the suggestion is to reject the study, a report providing the authors with specific points experimentally addressable will help in improving the manuscript and facilitate eventual resubmission to another journal. Finally, if the report is not comprehensive and the authors are invited to resubmit a revised version, this will result in multiple rounds of review with more concerns raised at subsequent rounds and delays in the publication process.

In an effort to reduce subconscious bias *Nature Communications* also offers the possibility to conceal authors' identities to the reviewers, so that the identity of the authors is initially known only to the editors. This double-blind peer review (http://dx.doi.org/10.1038/ncomms8774) scheme should help remove any potential or perceived bias arising from age, gender, nationality or the name of individual authors.

Last but not least, to facilitate the transparency of our editorial decisions, and to support post-publication discussion of our papers, we have launched a transparent peer review scheme (http://dx.doi.org/10.1038/ncomms10277). Here, authors can decide to publish reviewers' reports and the point-by point response files along with the accepted version of the manuscript. This process allows more transparency on the editorial process while still maintaining the reviewer's anonymity. The reviewers' identities are not disclosed unless they choose to sign their reports. Further information on the implementation and uptake of this scheme is discussed in a separate editorial at http://dx.doi.org/10.1038/ncomms13626.

With many of these initiatives being implemented or strengthened in the past years, we are continuingly revising the peer-review process working alongside the scientific community to provide the best service as possible to successfully disseminate solid, high-impact science.

Published online: 10 Nov 2016

